# LLM Adaptive PID Control for B5G Truck Platooning Systems

**DOI:** 10.3390/s23135899

**Published:** 2023-06-25

**Authors:** I. de Zarzà, J. de Curtò, Gemma Roig, Carlos T. Calafate

**Affiliations:** 1 Informatik und Mathematik, GOETHE-University Frankfurt am Main, 60323 Frankfurt am Main, Germany; dezarza@em.uni-frankfurt.de (I.d.Z.); roig@cs.uni-frankfurt.de (G.R.); 2 Departamento de Informática de Sistemas y Computadores, Universitat Politècnica de València, 46022 València, Spain; calafate@disca.upv.es; 3 Estudis d’Informàtica, Multimèdia i Telecomunicació, Universitat Oberta de Catalunya, 08018 Barcelona, Spain; 4 HESSIAN Center for AI (hessian.AI), 64293 Darmstadt, Germany

**Keywords:** platooning, coordination of vehicles, adaptive PID control, large language models, V2V communication, 5G and B5G systems

## Abstract

This paper presents an exploration into the capabilities of an adaptive PID controller within the realm of truck platooning operations, situating the inquiry within the context of Cognitive Radio and AI-enhanced 5G and Beyond 5G (B5G) networks. We developed a Deep Learning (DL) model that emulates an adaptive PID controller, taking into account the implications of factors such as communication latency, packet loss, and communication range, alongside considerations of reliability, robustness, and security. Furthermore, we harnessed a Large Language Model (LLM), GPT-3.5-turbo, to deliver instantaneous performance updates to the PID system, thereby elucidating its potential for incorporation into AI-enabled radio and networks. This research unveils crucial insights for augmenting the performance and safety parameters of vehicle platooning systems within B5G networks, concurrently underlining the prospective applications of LLMs within such technologically advanced communication environments.

## 1. Introduction and Related Work

The concept of truck platooning [1,2,3,4] is gaining significant attention due to its potential to improve fuel efficiency, reduce traffic congestion, and enhance road safety [5,6]. In a platoon, multiple trucks travel closely together, maintaining a constant distance to minimize air drag and save fuel. Adaptive PID (Proportional-Integral-Derivative) controllers play a crucial role in maintaining a constant inter-vehicle distance and ensuring the stability of the platoon. By adjusting the controller gains in real time based on the system’s behavior, adaptive PID controllers [7] can enhance the performance of the platoon and adapt to various driving conditions.

Effective communication between vehicles is fundamental for the successful implementation of truck platooning. Vehicle-to-vehicle (V2V) [8,9] communication enables trucks to share vital information, such as the speed, position, and braking status, with other vehicles in the platoon. This information is essential for maintaining a safe and constant distance between the trucks, which ensures an efficient platoon operation. The key aspects of communication that impact the performance of a platoon include the communication latency, packet loss, communication range, reliability, and robustness. Moreover, the security of the communication system is of the utmost importance, as it protects the platoon from potential cyberattacks and ensures the safety of the drivers and the cargo.

In recent years, the development of Cognitive Radio and AI-enabled 5G and Beyond 5G (B5G) networks has opened up new opportunities for advanced vehicular communication systems [10,11,12]. Indeed, one such application is truck platooning [13,14,15]. Our goal with this work is to emphasize the role of AI-enabled radio and networks in enhancing communication between vehicles, thereby addressing key challenges.

This paper presents an adaptive PID controller [16,17,18] that utilizes a Deep Learning (DL) model for efficient and reliable truck platooning. The controller is designed to handle various aspects of vehicle-to-vehicle (V2V) communication, such as communication latency, packet loss, communication range, reliability, and robustness. Furthermore, security concerns [19,20] are addressed to ensure the safety of the platoon.

**Figure 1:**
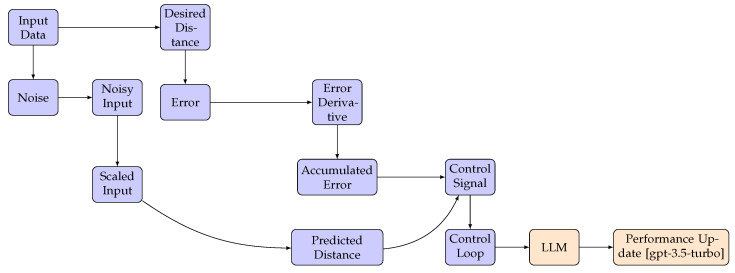
Flow diagram of the adaptive PID controller with LLM performance updates.

We begin with a base adaptive PID controller [21,22] that leverages a trained neural network model to predict the actual inter-vehicle distance. The controller is then improved by incorporating considerations relating to communication latency, packet loss, and communication range, as we believe in the importance of a reliable and robust communication system for the safe operation of the platoon. Moreover, a cutting-edge Large Language Model (LLM) [23,24,25], GPT-3.5-turbo, is integrated into the system to obtain real-time performance updates, demonstrating an innovative application of LLMs in the context of truck platooning. The results indicate that our adaptive PID controller, along with the LLM-based performance updates, offers a promising solution for efficient and secure truck platooning. An illustration of the proposed system is depicted in Figure 1.

This research aims to provide valuable insights into the design and implementation of AI-driven control systems for truck platooning in B5G networks while highlighting the potential of LLMs in advanced communication environments.

The core contributions of this study are twofold. Firstly, we put forth an adaptive PID controller, aimed at bolstering the efficiency and reliability in truck platooning within AI-enabled 5G and B5G network contexts. This controller deploys a DL model to forecast actual inter-vehicle distances and introduces factors such as communication latency, packet loss, communication range, and system reliability and robustness. These enhancements are designed to augment the performance and safety of the platoon amid varied driving conditions. Secondly, we introduce the integration of a cutting-edge Language Learning Model (LLM), GPT-3.5-turbo, into the control loop. The LLM provides real-time updates and recommendations, thereby augmenting the adaptability and explainability of the PID controller. This innovative usage of LLMs within the realm of truck platooning allows the system to tap into natural language comprehension abilities, which then leads to improved decision making and system optimization. Taken together, the adaptive PID controller and the LLM integration represent a comprehensive solution that guarantees the effective and secure truck platooning in B5G networks.

The remainder of this manuscript is organized as follows: Section 2 introduces the adaptive PID controller, emphasizing its critical role in control systems, with particular attention given to vehicle platooning. Section 3 outlines the methodology employed to design and implement the adaptive PID controller, addressing key considerations and challenges such as the latency, packet loss, communication range, noise channel, and security. Subsequently, Section 4 explores the integration of LLMs as a means to enhance the performance of the adaptive PID controller through real-time updates and recommendations. In Section 5, we delve into the potential implications of our research. Finally, Section 6 concludes the paper and outlines potential directions for future research.

## 2. Adaptive PID Controller

A Proportional-Integral-Derivative (PID) controller is a widely used control algorithm in various control systems. It calculates the control signal based on the error, the integral of the error, and its derivative. The error (e(t)) is the difference between the desired setpoint (r(t)) and the measured process variable (y(t)):

(1) e(t)=r(t)−y(t).

The control signal u(t) generated by the PID controller is given by the following equation:

(2) $$u\left(t\right)=K_{p}e\left(t\right)+K_{i}\int_{0}^{t}e\left(\tau \right)d\tau +K_{d}\frac{de(t)}{dt},$$

where $K_{p}$, $K_{i}$, and $K_{d}$ are the proportional, integral, and derivative gains, respectively.

In an adaptive PID controller, the gains $K_{p}$, $K_{i}$, and $K_{d}$ are adjusted in real time based on the system’s performance. The goal is to maintain optimal control performance despite changes in the system dynamics or external disturbances. Various methods exist for tuning the PID gains adaptively, such as Ziegler–Nichols [26], Cohen–Coon, and model-based approaches [16]. In this work, we employed a data-driven approach by training a deep neural network (DNN) to predict the optimal PID gains for the given system state. The DNN was trained on a synthetic dataset that captured a wide range of system behaviors and conditions and was used to illustrate its practical application. This allowed the adaptive PID controller to adjust the gains in real time based on the system’s current state, thus ensuring optimal control performance.

This paper primarily centers on the creation and assessment of an adaptive DNN-based PID control methodology for truck platooning. Although the synthetic dataset we utilized for experimentation did not overtly include vehicle model parameters such as mass, inertia, aerodynamic drag, tire friction, and powertrain features, it is crucial to stress that our approach can still be implemented when these parameters are either known or can be estimated. The synthetic dataset was representative, and its use demonstrated the efficacy and performance of our adaptive control method.

The adaptive PID controller employs the strength of deep neural networks to understand and adapt to the inherent dynamics of the truck platooning system. This enables it to effectively manage the changes in vehicle characteristics and driving conditions. This work propels the progression of adaptive control strategies within the scope of truck platooning, setting the stage for future research that could merge intricate vehicle models with parameter estimation methodologies.

The DNN used in this work comprised three types of layers: the input, hidden, and output layers. The input layer accepted the normalized system state, and the output layer generated the predicted PID gains. The hidden layers contained multiple neurons with activation functions, which facilitated the learning of complex nonlinear relationships between the input and output. Specifically, our DNN architecture included two hidden layers, the first with 64 neurons and the second with 32 neurons, both using the Rectified Linear Unit (ReLU) activation function. To prevent overfitting, we also incorporated dropout layers with a dropout rate of 0.2.

We adopted a supervised learning approach to train the DNN and utilized a synthetic dataset comprising various system states and their corresponding optimal PID gains. The dataset was generated using different parameters, such as desired and actual distances, vehicle speed, acceleration, road grade, and weather conditions. The dataset was then divided into training and testing sets, using an 80/20 split.

**Figure 2:**
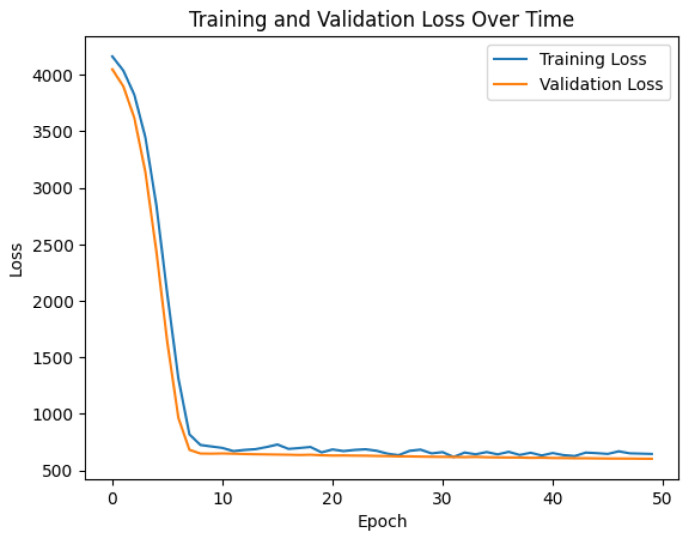
Training and validation loss over time for the proposed architecture.

To optimize the DNN, we minimized the mean square error (MSE) loss function, which measures the difference between the predicted PID gains and the true optimal gains. For this purpose, we employed the Adam optimizer with a learning rate of 0.001. The training process also included a validation split of 20%, with the model being trained for 50 epochs using a batch size of 32; the training and validation loss over time is shown in Figure 2. Once the DNN is trained, it can predict optimal PID gains for new unseen system states, enabling the adaptive PID controller to adjust its gains in real time and maintain optimal control performance.

**Figure 3:**
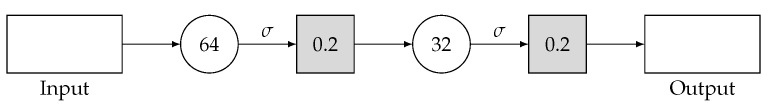
Detailed network architecture of the deep neural network (DNN) used for the adaptive PID tuning. The model consists of two fully connected layers with 64 and 32 neurons, followed by dropout layers with a rate of 0.2. Rectified Linear Unit (ReLU) activation functions, depicted as σ, are applied after each fully connected layer. The input and output layers are also depicted.

The trained DNN, shown in Figure 3, was integrated into the control loop of the truck platooning system. At each time step, the current system state was passed as input to the DNN, which predicted the optimal PID gains. These gains were then used to calculate the control signal, which adjusted the truck’s acceleration or deceleration to maintain a safe and constant inter-vehicle distance. This adaptive approach allows the PID controller to respond effectively to changes in the system dynamics and external disturbances, ensuring the stable and efficient operation of the platoon.

This study, while recognizing the importance of stability analysis, did not undertake a comprehensive formal stability examination via strict mathematical methods. We acknowledge that such an analysis is a fundamental facet of controller design, especially within the realm of adaptive control systems; the focus of our work, however, veered towards the implementation of LLMs for the sake of explainability.

Stability analysis in adaptive control systems typically involves an examination of the closed-loop system’s stability attributes, a task rendered complex by the controller’s adaptive properties. Traditional stability analysis methods, such as the Lyapunov stability theory and small-gain theorems, are usually employed to scrutinize the stability of adaptive control systems. These methods often require the establishment of suitable Lyapunov functions or the study of system gains to ensure stability and convergence.

The intricate nature and rigorous mathematical demand of a complete stability analysis meant that it was beyond the scope of our current work. Future research may seek to perform an in-depth stability analysis to provide formal assurances and further substantiate the stability properties of the adaptive PID controller under various scenarios. The main thrust of this paper, however, is to explore and highlight the utility of LLMs for the enhancement of system explainability.

## 3. Methodology

For our simulation, we utilized a platoon of two trucks that maintained a safe inter-vehicle distance. The synthetic dataset under study considered a desired distance between the two trucks to be within the range of 20–100 m (although in commercial applications, the range would be much lower in order to benefit from the aerodynamic drag), with their speeds varying between 40 and 120 km/h. The safe distance between the vehicles was determined based on various factors, such as the trucks’ speeds, acceleration, road grade, and weather conditions. The control loop calculated the control signal based on the current state, which was then used to update the truck’s acceleration or deceleration.

The choice of varying the desired distance between 20 and 100 m was meant to simulate different traffic scenarios and communication conditions that may affect the performance of the platoon control system. In real-world applications, the desired distance between vehicles might not necessarily remain constant, as factors such as traffic density, road conditions, and safety considerations can influence the optimal distance.

To simulate the communication latency, we modified the control loop to include a circular buffer for the control signals. This buffer represents the delay in communication between vehicles, with each element in the buffer corresponding to a time step of latency.

**Figure 4:**
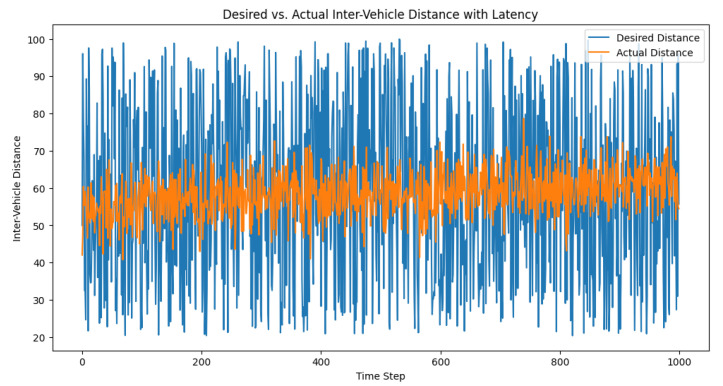
Desired vs. actual inter-vehicle distance with latency.

**Figure 5:**
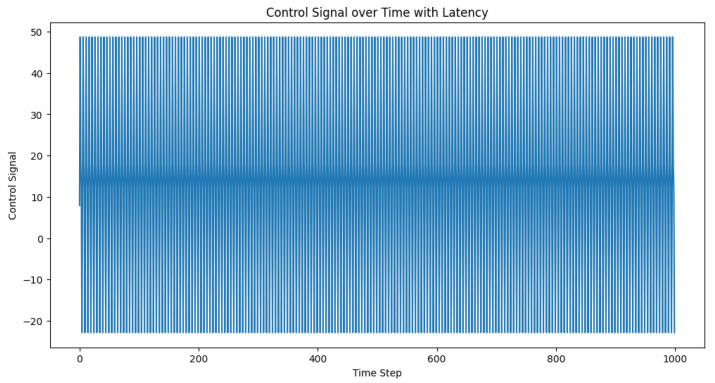
Temporal evolution of the control signal amid the latency. The control signal encapsulates the system modifications applied to sustain the requisite distance between the vehicles within a truck platoon, acting as a responsive adjustment to the latency-induced variations.

We set the communication latency (in time steps) and initialized the control signal buffer accordingly. The desired distances, actual distances, and control signals were recorded for each time step. After running the simulation, we visualized the desired and actual inter-vehicle distances, as well as the control signals over time. The plots in Figure 4 and Figure 5 can help in the analysis of the latency’s impact on the performance of the adaptive PID controller and its ability to maintain safe inter-vehicle distances.

Thus, communication latency was integrated into the control loop by simulating a circular buffer for the control signals. This modification, which meant that the effects of latency were now incorporated into the control loop, allowed us to analyze the performance of the adaptive PID controller under various latency conditions. In the control loop, the buffer was used to store and retrieve control signals with the specified latency. At the beginning of each iteration, a placeholder was added to the buffer, and the delayed control signal was retrieved by popping the first element. If the delayed control signal was available, it was used to control the vehicle; otherwise, the current PID calculation was used. This process simulated communication latency and helped us to understand its impact on the system’s performance. In this specific example, a latency of five time steps was used.

**Figure 6:**
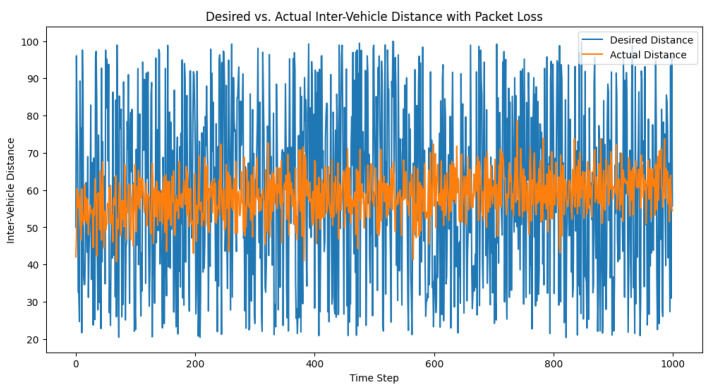
Desired vs. actual inter-vehicle distance with packet loss.

**Figure 7:**
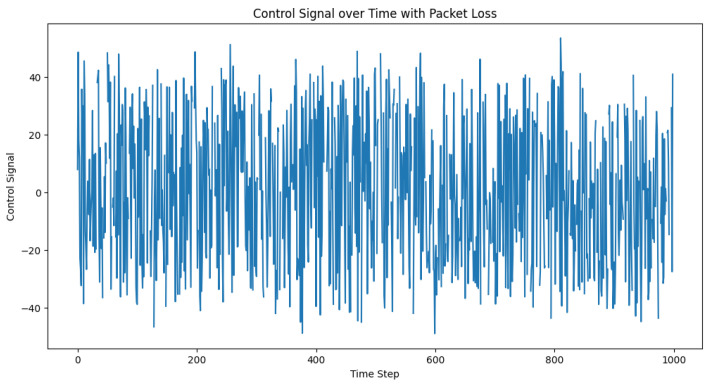
Control signal trajectory amid the packet loss. This illustrates the control signal’s role as a corrective mechanism that dynamically adjusts to maintain the intended inter-vehicle distance within a truck platoon, demonstrating its resilience despite the packet loss events.

To simulate the packet loss in the communication between vehicles, we modified the control loop to incorporate a packet loss rate. This rate represents the percentage of control signals that are lost during transmission. The packet loss was simulated by randomly setting the percentage of control signals to None based on the packet loss rate. Mathematically, we can define the packet loss rate as p∈[0,1], where p=0 means no packet loss, and p=1 means 100% packet loss. We then generated a random number r∈[0,1] for each time step, and if r<p, we set the control signal to None. We subsequently set the packet loss rate and ran the simulation, recording the desired distances, actual distances, and the control signals for each time step. After running the simulation, we visualized the desired and actual inter-vehicle distances, as well as the control signals over time. The plots in Figure 6 and Figure 7 can help in analyzing the impact of packet loss on the performance of the adaptive PID controller and its ability to maintain safe inter-vehicle distances.

In the specific example, the packet loss rate was set to 0.1 (or 10%). The code can also be used to test a range of packet loss rates to understand the sensitivity of the controller’s performance under different packet loss scenarios.

**Figure 8:**
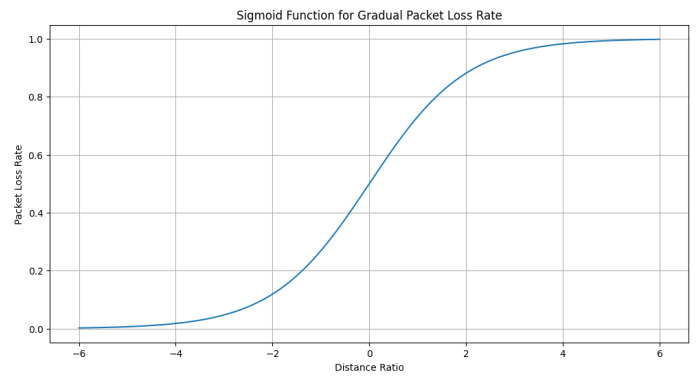
Sigmoid function for gradual packet loss rate. This plot illustrates the relationship between the distance ratio (predicted distance divided by communication range) and the packet loss rate. The sigmoid function demonstrates a gradual increase in packet loss rate as the distance ratio increases, simulating a more realistic communication scenario in the control loop.

**Figure 9:**
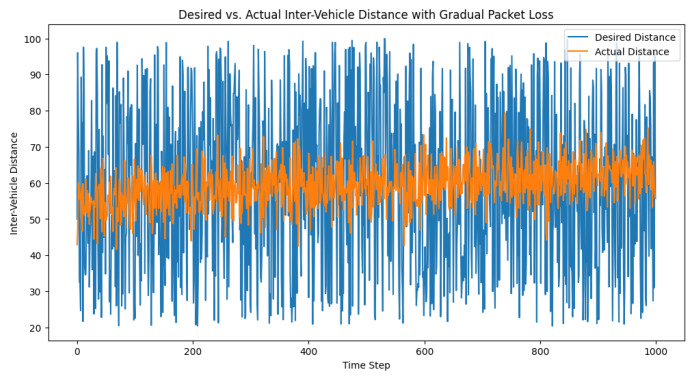
Desired vs. actual inter-vehicle distance with a gradual packet loss.

**Figure 10:**
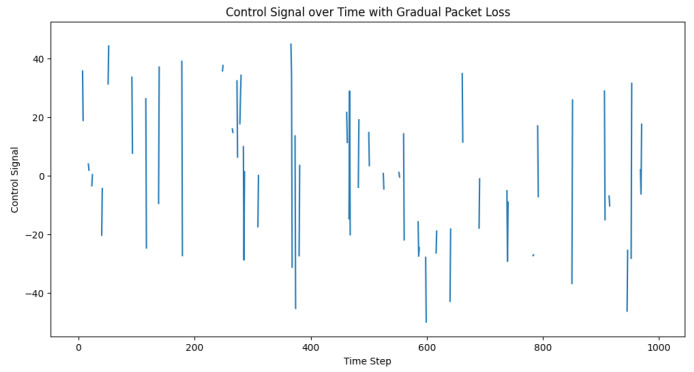
Temporal progression of the control signal amid gradual packet loss. The control signal functions as an adaptive mechanism that continually adjusts to preserve the targeted distance between vehicles within a truck platoon, even when confronting the challenges of a gradual packet loss.

However, in a real environment, the reality is that packet loss gradually increases as the distance increases, up until it reaches 100%. The all-or-nothing approach used in the previous code might not accurately represent this behavior. To better simulate the real-world scenario, we modified the control loop to incorporate a gradual increase in packet loss as the distance increased. Our approach involved the use of a sigmoid function, illustrated in Figure 8, to map the distance to a packet loss rate (see Figure 9 and Figure 10).

In our study, we introduced the gradual packet loss mechanism based on the sigmoid function to simulate a more realistic scenario where packet loss increases as the inter-vehicle distance approaches the maximum communication range. Although the sigmoid function indeed resulted in a 100% packet loss rate when the maximum communication range was reached, it was essential to conduct experiments in order to investigate the system’s behavior under varying communication conditions and packet loss rates.

The purpose of these experiments was to demonstrate the performance and robustness of the proposed control strategy in maintaining the desired inter-vehicle distance despite the presence of communication challenges. Figure 9, which presents the distances between trucks, may not show significant differences as compared to the previous results. However, it is crucial to highlight the controller’s capability to handle communication limitations and maintain satisfactory performance even when the vehicles were close to or at the communication range’s limits. This observation underscores the importance of analyzing the impact of communication range and packet loss on the control system’s performance in real-world applications.

**Figure 11:**
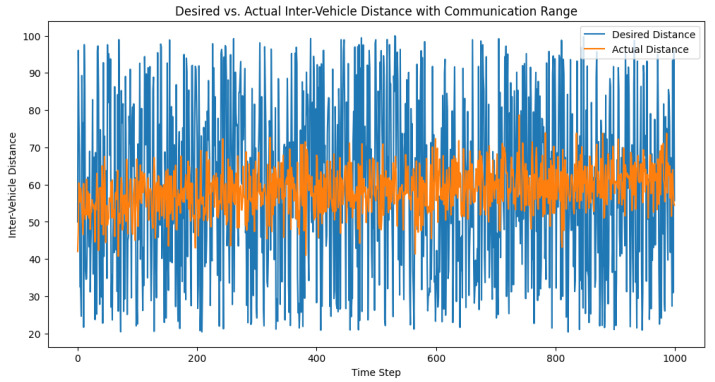
Desired vs. actual inter-vehicle distance with the communication range.

**Figure 12:**
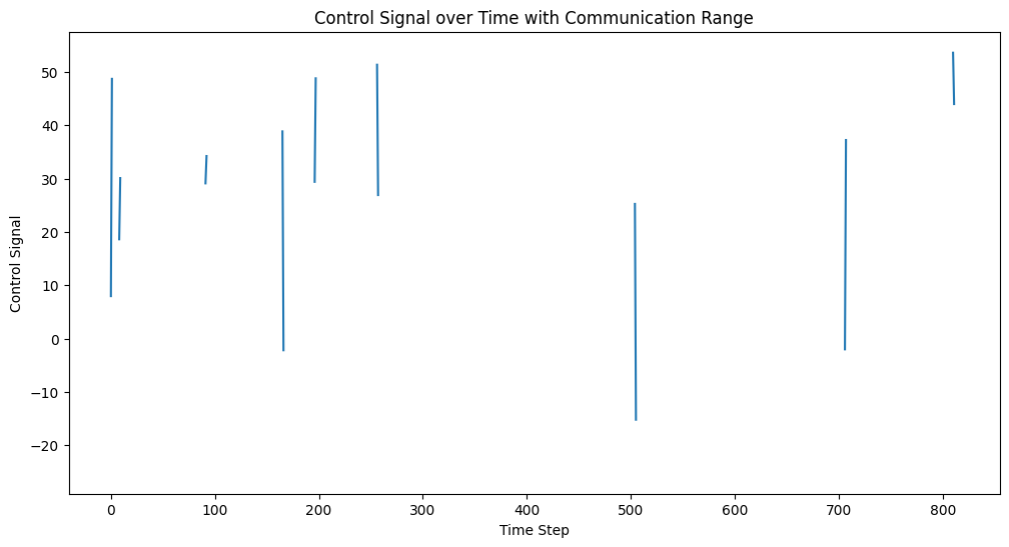
Control signal trajectory in varying communication ranges. The control signal, depicted here, acts as a real-time corrective measure that effectively regulates inter-vehicle distance within a truck platoon, demonstrating its adaptability across different communication range scenarios.

In order to simulate the effect of communication range limitations on the adaptive PID controller, we modified the control loop to take into account the communication range. When the predicted inter-vehicle distance was greater than the communication range, the control signal was set to None, which simulated a lack of communication between the vehicles. We then set the communication range and ran the simulation, recording the desired distances, actual distances, and control signals for each time step. After running the simulation, we visualized the desired and actual inter-vehicle distances, as well as the control signals over time. The plots in Figure 11 and Figure 12 help in analyzing the impact of communication range limitations on the performance of the adaptive PID controller and its ability to maintain safe inter-vehicle distances.

**Figure 13:**
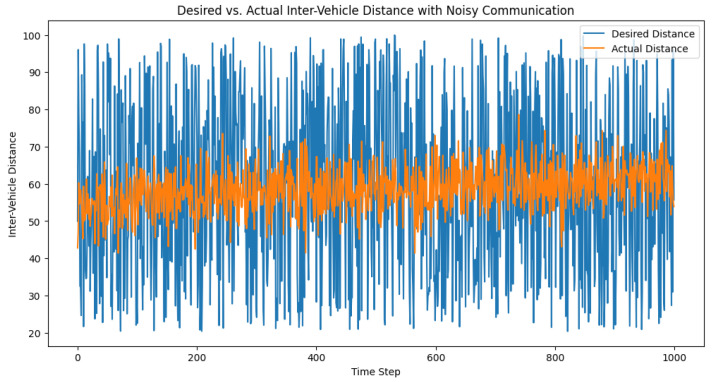
Desired vs. actual inter-vehicle distance with noisy communication.

**Figure 14:**
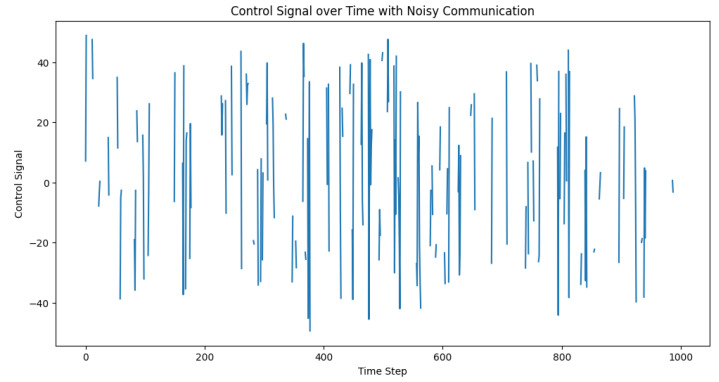
Control signal behavior amid noisy communication. This depiction of the control signal underscores its role as a dynamic corrective measure, adjusting in real time to manage inter-vehicle distances within a truck platoon, even under the challenging conditions of communication noise.

To evaluate the effect of noisy communication on the adaptive PID controller, we modeled the impact of Gaussian noise on the packet loss, which influences the system’s ability to accurately calculate the control signal. Let $N(0,\sigma^{2})$ be the Gaussian noise, with mean 0 and standard deviation σ. We incorporated the noise effect by mapping the noise standard deviation to a packet loss rate using a sigmoid function. We then ran the control loop with the noise-affected packet loss rate and recorded the desired distances, actual distances, and control signals for each time step. After running the simulation, we visualized the desired and actual inter-vehicle distances, as well as the control signals over time. The plots in Figure 13 and Figure 14 help in analyzing the performance of the adaptive PID controller under noisy communication conditions and its ability to maintain safe inter-vehicle distances.

The reason behind adding Gaussian noise to the packet loss rate is the following: While it is true that the sigmoid function models the effect of signal attenuation on packet loss, we wanted to explore the effect of other sources of noise that could also impact the communication range, such as atmospheric conditions or interference from other wireless signals. By adding Gaussian noise to the packet loss rate, we introduced a random component to the simulation that could help us better understand the robustness of the platooning system to different sources of noise. Moreover, the analysis of the probability distribution function of the distance could also provide us with a better understanding of the behavior of the platoon system under different noise conditions.

In the context of the control signal plot, the values displayed represent u(t), which was the control signal at each time step t. The control signal u(t) was calculated based on the PID controller’s output, which combined the proportional, integral, and derivative terms of the error between the desired distance and the actual distance. The purpose of the control signal is to adjust the behavior of the following vehicle in the platoon in order to minimize the error and maintain the desired inter-vehicle distance.

In the presence of packet loss or communication limitations, some control signals might be lost, which could lead to fluctuations in the actual distance as the controller tries to compensate for the missing information. The control signal plot visualizes the u(t) values over time, allowing for the assessment of the system’s performance and robustness in the face of communication challenges.

In this scenario, the potential impact of the secure communication between vehicles on the adaptive PID controller is considered. It should be noted that encryption is not typically used at the physical layer in vehicular networks, and the primary impact of encryption in this context would be the added delay introduced by the encryption and decryption process. However, for completeness, a demonstration of how one might implement encrypted communication using the Advanced Encryption Standard (AES) for symmetric encryption with the Python cryptography library is provided as follows.

- encrypt_data(data, key, iv): encrypts the given data using the provided key and initialization vector (IV) with the AES in CBC mode and PKCS7 padding;
- decrypt_data(encrypted_data, key, iv): decrypts the given encrypted data using the provided key and initialization vector (IV) with the AES in CBC mode and PKCS7 padding.

In the control loop, the following steps were performed:

1. Calculate the control signal as usual;
2. Encrypt the control signal using encrypt_data() with a randomly generated AES key and IV;
3. Transmit the encrypted control signal. In a real system, this would involve sending the data between vehicles;
4. Decrypt the received encrypted control signal using decrypt_data() with the same AES key and IV;
5. Continue with the decrypted control signal in the control loop.

**Figure 15:**
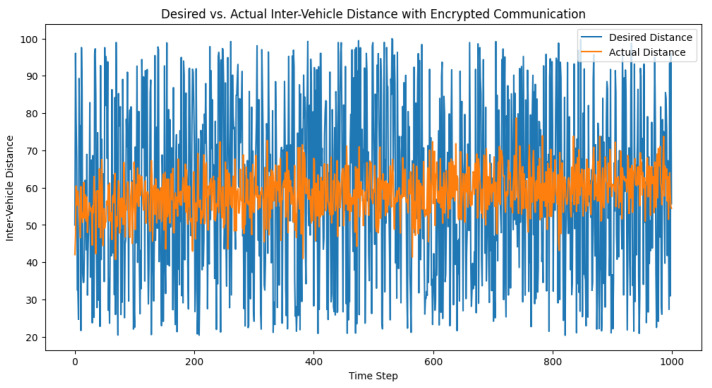
Desired vs. actual inter-vehicle distance with encrypted communication.

**Figure 16:**
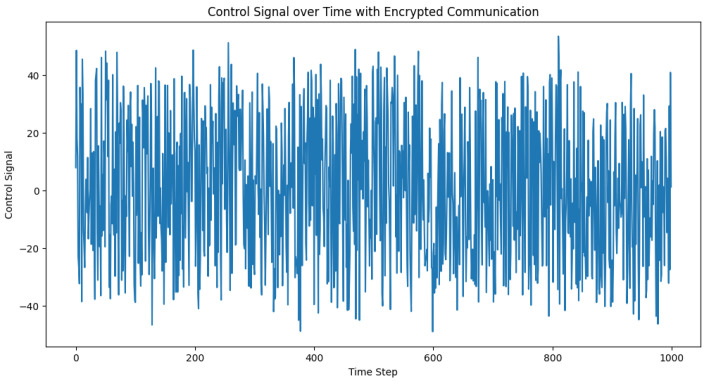
Control signal over time with encrypted communication. The control signal represents the adjustment applied to the system to maintain the desired distance between the vehicles in a truck platoon.

The primary takeaway from this exercise is the potential impact of the additional latency that was introduced by the encryption and decryption process. The simulation results may not significantly differ from the unencrypted scenario, as depicted in Figure 15 and Figure 16, as the added delay from encryption and decryption was not incorporated into this demonstration. In practice, the delay should be taken into account when analyzing the performance of the adaptive PID controller.

## 4. Integration with Large Language Models

Large Language Models (LLMs) [27,28,29,30] are advanced ML models trained on vast amounts of text data. These models have achieved state-of-the-art results in various natural language understanding tasks, including text generation, translation, summarization, and question answering. LLMs are capable of understanding the context, generating coherent responses, and providing valuable insights based on the data they are exposed to. In this section, we explore the integration of LLMs into a control loop system and demonstrate their potential to enhance the system’s performance.

The GPT-3.5-turbo is built on the Transformer architecture, which was first introduced in [31]. The architecture employs self-attention mechanisms [32] that enable the model to process and understand long-range dependencies in the input text. Mathematically, the self-attention mechanism can be expressed as follows:

(3) $$\mathrm{Attention}\left(Q,K,V\right)=\mathrm{softmax}\left(\frac{QK^{T}}{\sqrt{d_{k}}}\right)V,$$

where *Q*, *K*, and *V* represent the query, key, and value matrices, respectively, and $d_{k}$ is the dimension of the key vectors. The softmax function normalizes the attention scores and helps the model to focus on the most relevant parts of the input text.

The training process involves optimizing the model’s parameters in order to minimize the cross-entropy loss between the predicted token probabilities and the actual target tokens in a given context. This was carried out using the synthetic dataset that we generated. The loss function can be expressed as follows:

(4) $$L\left(\theta \right)=-\sum_{t=1}^{T}\log p\left(y_{t}|y_{1:t-1},x;\theta \right),$$

where L(θ) is the loss function, $y_{t}$ is the target token at time step *t*, x is the input context, and θ represents the model’s parameters.

During inference, the LLM generates the text by sampling from the probability distribution over the vocabulary. The model employs a temperature parameter (τ) to control the randomness of the generated text. Lower values of τ result in more deterministic outputs, whereas higher values increase the diversity of the generated text. The probability of selecting a token $y_{t}$ at time step *t* can be defined as follows:

(5) $$p\left(y_{t}|y_{1:t-1},x;\theta ,\tau \right)=\frac{\exp(f_{\theta }\left(y_{1:t-1},x\right)/\tau )}{\sum_{y^{\prime }t}\exp\left(f\theta \left(y_{1:t-1}^{\prime },x\right)/\tau \right)},$$

where $f_{\theta }$ is the model’s prediction function.

In the following example, we use a control loop system designed to maintain a desired inter-vehicle distance in an autonomous vehicle. The control loop’s primary function is to calculate the control signals that help the vehicle maintain the desired distance. The system employs an adaptive PID controller that predicts the distance based on input data, calculates the error, and adjusts the control signals accordingly.

The control loop system operates in a noisy communication environment, simulating real-world scenarios, where input data can be affected by noise. The code snippet below defines the control loop with noise:

(6) input_data_noisy=input_data+N(0,noise_stddev),

where N(0,noise_stddev) represents the Gaussian noise with mean 0 and standard deviation noise_stddev.

The control loop calculates the error between the desired and predicted distances:

(7) error=desired_distance−predicted_distance.

Using proportional ($K_{p}$), integral ($K_{i}$), and derivative ($K_{d}$) gains, the control signal is calculated as follows:

(8) $$\begin{array}{c} \mathrm{control}\_\mathrm{signal}=K_{p}\cdot \mathrm{error}+\\ K_{i}\cdot \mathrm{accumulated}\_\mathrm{error}+K_{d}\cdot \mathrm{error}\_\mathrm{derivative}. \end{array}$$

LLMs can be integrated into the control loop to provide performance updates and insights at specific intervals, as depicted in the flow diagram in Figure 1. In this example, the LLM is queried every 100 time steps. The LLM receives the input data string and returns a performance update.


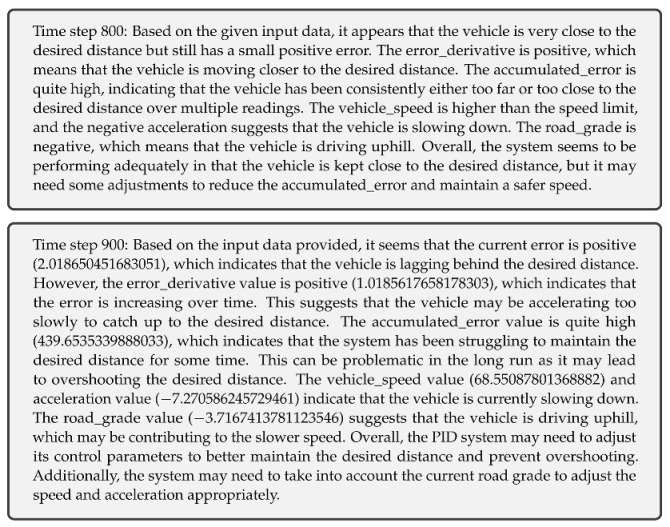


These updates can help engineers analyze the performance of the control loop and potentially suggest improvements or detect issues. Integrating Large Language Models into control loop systems can provide valuable insights, performance updates, and support when optimizing the system’s performance. The example provided demonstrates how LLMs can be effectively used in conjunction with an adaptive PID controller in a noisy communication environment. This approach opens up new possibilities for leveraging the power of LLMs in various control applications across different domains.

## 5. Discussion

In this study, we delved into the integration of LLMs, particularly the GPT-3.5-turbo, into the control loop of a convoy of autonomous vehicles. We demonstrated the potential of LLMs to bolster the control loop performance by providing immediate feedback and recommendations. Furthermore, we scrutinized the impact of variables such as noisy communication, encryption, latency, packet loss, and communication range on the performance of the system, underlining the importance of secure and reliable communication for safety-critical applications.

An area of promising potential application for our findings is in the domain of unmanned aerial vehicles (UAVs), or drones. Similar to autonomous vehicles, drones require sophisticated control mechanisms to ensure stable flight and efficient route planning. PID controllers are a crucial component in drone flight systems as they are responsible for achieving and maintaining the drone’s balance and orientation based on sensory input. The integration of LLMs could provide additional layers of interpretability and adaptability to these systems, potentially leading to safer and more reliable drone operations.

Our experiments demonstrated that the amalgamation of LLMs and conventional control techniques can indeed enhance the performance of complex systems such as autonomous vehicle platoons. LLMs provide new opportunities to leverage their natural language understanding capabilities for a plethora of applications, including diagnostics, decision making, and real-time system optimization. Our research accentuates the potential of LLM integration across an extensive range of engineering domains, where they can supplement and augment conventional control techniques.

While the use of synthetic data enabled us to demonstrate the virtues of our control scheme within a controlled environment, we acknowledge the necessity for further validation using real-world data. The focus of this paper was the innovative combination of an adaptive PID controller with an LLM to enhance explainability. Therefore, we were primarily concentrated on the theoretical framework and its potential implementations.

## 6. Conclusions and Future Work

This study offered valuable insights into the design and implementation of AI-driven control systems for truck platooning within B5G networks and showcased the promising potential of LLMs in advanced telecommunication environments. Future work should aim to utilize actual data from real-world truck platooning systems, which could thereby provide a rigorous evaluation of our proposed control strategy.

Future research directions for LLMs could include the following:

- Assessment of forthcoming LLM architectures and the training methodologies’ impact on the efficiency of adaptive control systems;
- Development of methods for the real-time fine-tuning of LLMs, allowing for swift adaptation to dynamic environments;
- Exploration of LLM applicability to other safety-critical domains such as aerospace and medical systems;
- Advancement of LLM-based control strategies in multiagent systems;
- Investigation of the combination of LLMs with alternative AI approaches, such as reinforcement learning.

For the adaptive PID control, future research could entail the following:

- Extension of the framework to cater to complex systems with nonlinear dynamics;
- Examination of the performance of different RL algorithms, such as Q-Learning or Actor–Critic, for the tuning of the PID controller gains;
- Development of hybrid control strategies that combine adaptive PID control with other control approaches;
- Integration of advanced sensing and communication technologies, such as LiDAR or V2X communication;
- Exploration of the integration of multiple control systems, such as a multiagent system, for larger-scale control problems.

## Data Availability

Not applicable.

## Abbreviations

The following abbreviations are used in this manuscript:

LLM	Large Language Models
PID	Proportional-Integral-Derivative
DL	Deep Learning
DNN	Deep Neural Networks
ML	Machine Learning
V2V	Vehicle-to-Vehicle
GPT	Generative Pretraining Transformer
AES	Advanced Encryption Standard
